# Sirtuin 3 regulates mitochondrial protein acetylation and metabolism in tubular epithelial cells during renal fibrosis

**DOI:** 10.1038/s41419-021-04134-4

**Published:** 2021-09-13

**Authors:** Yu Zhang, Ping Wen, Jing Luo, Hao Ding, Hongdi Cao, Weichun He, Ke Zen, Yang Zhou, Junwei Yang, Lei Jiang

**Affiliations:** 1 Center for Kidney Disease, The second Affiliated Hospital, Nanjing Medical University, Nanjing, Jiangsu 210003 China; 2 State Key Laboratory of Pharmaceutical Biotechnology, Nanjing University Advanced Institute of Life Sciences, Nanjing, Jiangsu 210093 China

**Keywords:** Protein-protein interaction networks, End-stage renal disease

## Abstract

Proximal tubular epithelial cells (TECs) demand high energy and rely on mitochondrial oxidative phosphorylation as the main energy source. However, this is disturbed in renal fibrosis. Acetylation is an important post-translational modification for mitochondrial metabolism. The mitochondrial protein NAD^+^-dependent deacetylase sirtuin 3 (SIRT3) regulates mitochondrial metabolic function. Therefore, we aimed to identify the changes in the acetylome in tubules from fibrotic kidneys and determine their association with mitochondria. We found that decreased SIRT3 expression was accompanied by increased acetylation in mitochondria that have separated from TECs during the early phase of renal fibrosis. *Sirt3* knockout mice were susceptible to hyper-acetylated mitochondrial proteins and to severe renal fibrosis. The activation of SIRT3 by honokiol ameliorated acetylation and prevented renal fibrosis. Analysis of the acetylome in separated tubules using LC–MS/MS showed that most kidney proteins were hyper-acetylated after unilateral ureteral obstruction. The increased acetylated proteins with 26.76% were mitochondrial proteins which were mapped to a broad range of mitochondrial pathways including fatty acid β-oxidation, the tricarboxylic acid cycle (TCA cycle), and oxidative phosphorylation. Pyruvate dehydrogenase E1α (PDHE1α), which is the primary link between glycolysis and the TCA cycle, was hyper-acetylated at lysine 385 in TECs after TGF-β1 stimulation and was regulated by SIRT3. Our findings showed that mitochondrial proteins involved in regulating energy metabolism were acetylated and targeted by SIRT3 in TECs. The deacetylation of PDHE1α by SIRT3 at lysine 385 plays a key role in metabolic reprogramming associated with renal fibrosis.

## Introduction

Chronic kidney disease (CKD) is characterized by progressive kidney dysfunction of at least three months duration, and it affects about 10% of adults worldwide [1, 2]. Several pathologies including diabetes nephropathy (DN), chronic glomerulonephritis, hypertension, chronic tubulointerstitial nephritis, and polycystic kidney diseases (PKD) [3] lead to CKD. Regardless of the contributing pathology, irreversible fibrosis is the main feature of kidney failure in CKD [4, 5]. Extensive investigation has confirmed that renal interstitial fibrosis is not only a common histomorphological change associated with end-stage renal disease (ESRD) but also a key factor in determining renal progressive failure in CKD. Therefore, the pathogenesis of renal interstitial fibrosis has become a hot topic in the field of renal diseases.

Proximal tubular epithelial cells (TECs) are abundant in mitochondria and depend on fatty acid oxidation (FAO) and mitochondrial oxidative phosphorylation (OXPHOS) as energy sources to maintain the structure and functions of TECs, including excretion, secretion, and reabsorption [6, 7]. Mitochondrial homeostasis and energy metabolism in TECs play central roles in the development of renal disease as interstitial fibrosis progresses [7–9]. Recent studies have shown that the main causes of interstitial fibrosis are defective FAO and OXPHOS [10, 11]. Defective FAO in TECs is often associated with metabolic reprogramming to glycolysis during the progression of acute kidney injury (AKI), PKD, and DN [12–17]. However, the mechanism of metabolic reprogramming in TECs during the progression of renal fibrosis remains unclear.

N-lysine acetylation is a post-translational modification of histones that have been widely studied in transcriptional regulation. Proteomic analyses over the past decade have shown that non-histone proteins are frequently acetylated and are a major component of acetylation in mammalian cells. Non-histone acetylation is involved in various physiological processes, such as gene transcription, DNA damage and repair, cell division, signal transduction, protein folding, autophagy, and metabolism. Sirtuins are NAD^+ ^-dependent deacetylases involved in metabolism, stress response, and longevity. Among them, SIRT3 is a mitochondrial deacetylase that mediates the activity of many metabolic enzymes involved in the mitochondrial electron transport chain (ETC), TCA cycle, glycolysis, fatty acid metabolism, and ATP synthesis. Roles of SIRT3 have been identified in AKI [18–20], DN [14, 21–23], aging [21, 24, 25], and kidney tumors [26]. The role of SIRT3 in acetylation in the kidneys is mainly based on the acetylomes of organs such as the liver, and muscle. A quantitative map of the acetylome is required to understand the diverse mechanisms of acetylation in kidneys during the progress of renal fibrosis.

Here, we describe changes in the acetylome profile of tubules in fibrotic kidneys. Further, we identified altered acetylated proteins in tubules from fibrotic kidneys and determined their association with mitochondria. We hypothesized that SIRT3 is a major regulator of mitochondrial acetylation and metabolic homeostasis. Our findings will further our understating of the role of SIRT3 in metabolic reprogramming associated with renal fibrosis.

## Results

### Identification of SIRT3 regulated acetylation in human CKD

**Fig. 1:**
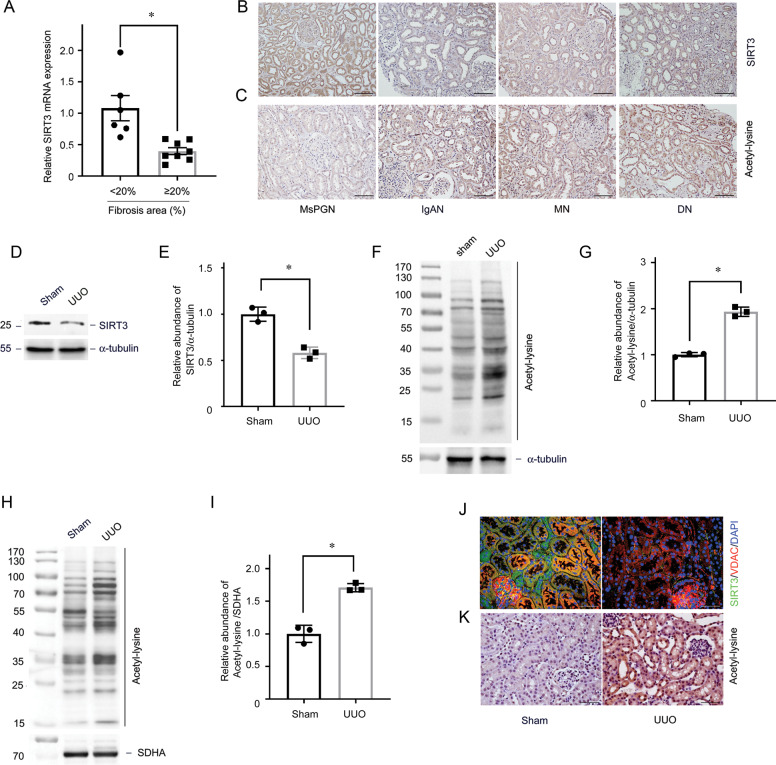
SIRT3 regulates acetylation of tubular mitochondrial proteins in renal fibrosis. **A** Relative *SIRT3* mRNA levels in human kidney sections. *P < 0.05 compared with fibrotic area <20%, *n* = 6–8. **B** Representative expression and localization of SIRT3 in various types of human CKD. **C** Representative immunohistochemical staining for acetylated lysine in kidney tissues from human CKD. The black bar indicates 100 μm (MsPGN mesangial proliferative glomerulonephritis, IgAN IgA nephropathy, MN membranous nephropathy, DN diabetic nephropathy). **D**, **E** Western blots of SIRT3 expression in fibrotic kidneys compared with sham on a postoperative day (POD) 1 (**D**). Relative abundance of SIRT3 expression, α-tubulin served as the standard (*P < 0.05; *n* = 3) (**E**). **F**, **G** Western blots of proteins from tubules isolated from kidneys of UUO and sham-operated mice at day 1 using an anti-acetylated-lysine antibody. α-tubulin served as the standard (*P < 0.05; *n* = 3). **H**, **I** Western blots of acetylated lysine in tubular mitochondrial lysates from sham-operated and UUO mice on POD 1. SDHA served as the standard (*P < 0.05; *n* = 3). **J**, **K** Representative immunostaining and immunohistochemical findings of SIRT3 and acetyl-lysine expression in kidneys. The bar indicates 50 μm.

We analyzed SIRT3 expression in kidney biopsy specimens from 14 patients with CKD and various degrees of renal fibrosis using quantitative real-time polymerase chain reaction (qRT-PCR) (Supplementary Table 1). The *SIRT3* mRNA level was decreased in the hyper-fibrosis group with the area of matrix deposition over 20% analysis by Masson staining compared with the hypo-fibrosis group with the area of matrix deposition below 20% (Fig. 1A). We also evaluated SIRT3 expression and acetylation profiles by immunostaining. Figure 1B shows that SIRT3 protein was predominantly localized in renal TECs in normal kidneys, but decreased in fibrotic areas in kidney biopsies from patients with a variety of CKD. Furthermore, the amount of acetylation was low in normal kidneys and increased in samples of fibrotic kidneys (Fig. 1C).

### Loss of SIRT3 leads to mitochondrial protein hyperacetylation in tubules in renal fibrosis

We created C57BL/6J mice with unilateral ureteral obstruction (UUO) to assess the role of SIRT3 in renal fibrosis. The mRNAs level of sirtuins were all decreased in tubules from mice with UUO on postoperative day (POD) 1 (Supplementary Fig. 1A). The decrease of *Sirt3* mRNA was most significant. Western blot analysis showed that the expression of SIRT3 was decreased in a time-dependent manner along with increased fibronectin (FN) and collagen I expression after UUO (Supplementary Fig. 1B, Fig. 1D, E), resulting in global elevated protein acetylation (Fig. 1F, G). Because SIRT3 is a mitochondrial deacetylase, we isolated mitochondria from tubules after UUO. Increased acetylation of mitochondrial proteins were detected from tubules from mice with UUO (Fig. 1H, I, Supplementary Fig. 1C). Immunostaining further confirmed decreased SIRT3 and increased acetylation in mitochondria (Fig. 1J, K, Supplementary Fig. 1D).

**Fig. 2:**
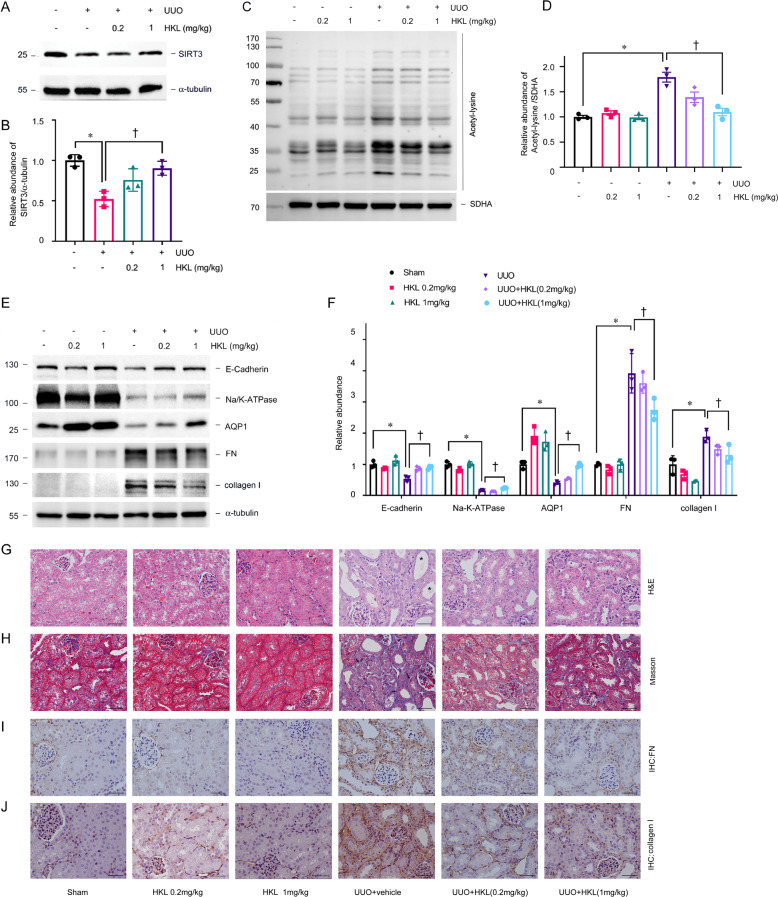
Honokiol reduces mitochondrial acetylation and renal fibrosis. **A**, **B**: Amount of SIRT3 protein in kidney tissues from UUO mice injected with or without honokiol (HKL). α-tubulin served as the standard (*P < 0.05, ^†^*P* < 0.05; *n* = 3). **C**, **D** Western blots of acetyl-lysine in tubular mitochondrial lysates from UUO mice injected with or without HKL on POD 1. SDHA served as the standard (*P < 0.05, ^†^*P* < 0.05; *n* = 3). **E**, **F** Western blots of E-cadherin, Na/K-ATPase, AQP1, FN, and collagen I in UUO mice injected with or without HKL on POD 7. α-tubulin served as the standard (*P < 0.05, ^†^*P* < 0.05; *n* = 3). **G**–**J** Representative images of H&E (**G**) (Star indicated tubular atrophy), Masson (**H**) (Arrow indicated collagen deposition), and immunohistochemical staining for FN (**I**) and collagen I (**J**) in kidneys on POD 7 after UUO in mice injected with honokiol. The bar indicates 50 μm.

**Fig. 3:**
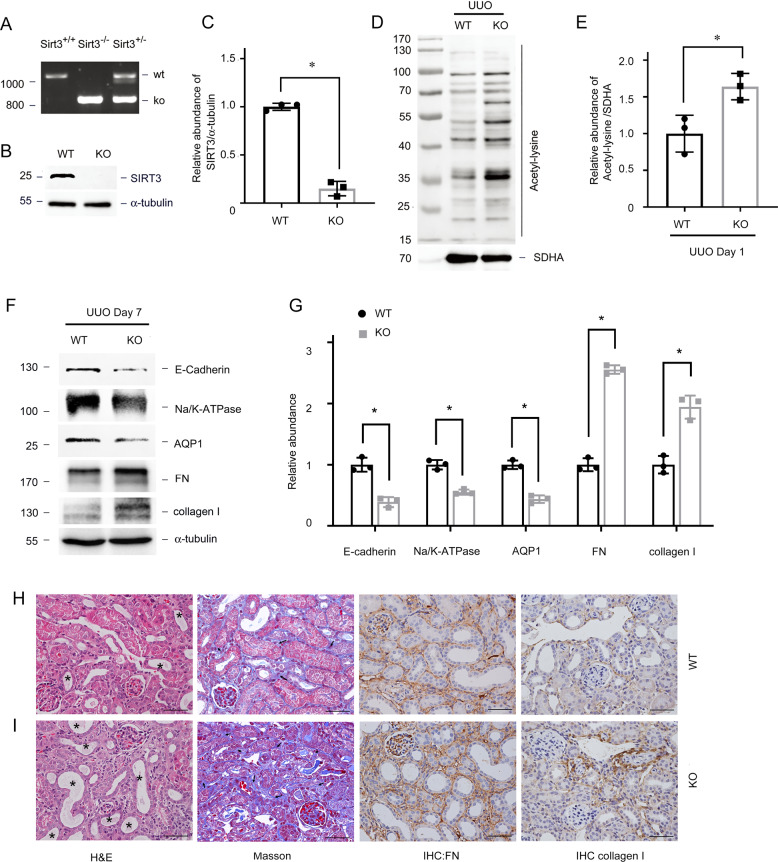
Ablation of SIRT3 aggravates renal fibrosis. **A** PCR findings of mouse genomic DNA. **B**, **C** Western blots of SIRT3 expression in kidneys of *Sirt3* KO and WT mice. α-tubulin served as the standard (*P < 0.05; *n* = 3). **D**, **E** Western blots of acetylated lysine in tubular mitochondrial lysates from *Sirt3* KO and WT mice with UUO on POD 1. SDHA served as the standard (*P < 0.05; *n* = 3). **F**, **G**: Representative immunoblots of E-cadherin, Na/K-ATPase, AQP1, FN, and collagen I in *Sirt3* KO and WT mice after UUO on POD 7. α-tubulin served as the standard (*P < 0.05; *n* = 3). **H**, **I** Representative images of H&E, Masson, and immunohistochemical staining for FN and collagen I in kidneys on POD 7 after UUO in *Sirt3* KO mice and WT mice (star indicated tubular atrophy; arrow indicated collagen deposition).

We treated C57BL/6J male mice with honokiol (HKL), an activator of SIRT3, to determine the role of SIRT3 in renal fibrosis [27, 28], and assessed changes in acetylation and metabolism in mitochondria. HKL restored SIRT3 expression, and significantly reduced mitochondrial acetylation in tubules (Fig. 2A–D). We then investigated whether SIRT3 activation correlates with tubular damage and renal fibrosis. HKL preserved the expression of proximal tubular cell marker including Na/K-ATPase, aquaporin 1 (AQP1), and E-cadherin, attenuated tubular atrophy, and reduced the accumulation of extracellular matrix proteins indicated by collagen I, FN expression, induced by UUO (Fig. 2E–J). We further analyzed mitochondrial acetylation profiles in *Sirt3* knockout (KO) mice (Fig. 3A–C). *Sirt3* KO did not affect the expression of other SIRTs (Supplementary Fig. 2A). The amount of acetylation in mitochondria was mildly increased in *Sirt3* KO mice (Supplementary Fig. 1B). However, *Sirt3* KO mice developed obvious mitochondrial acetylation and severe fibrosis after UUO in contrast to wild-type (WT) control littermates (Fig. 3D–I).

### SIRT3 regulates mitochondrial acetylation in vitro

**Fig. 4:**
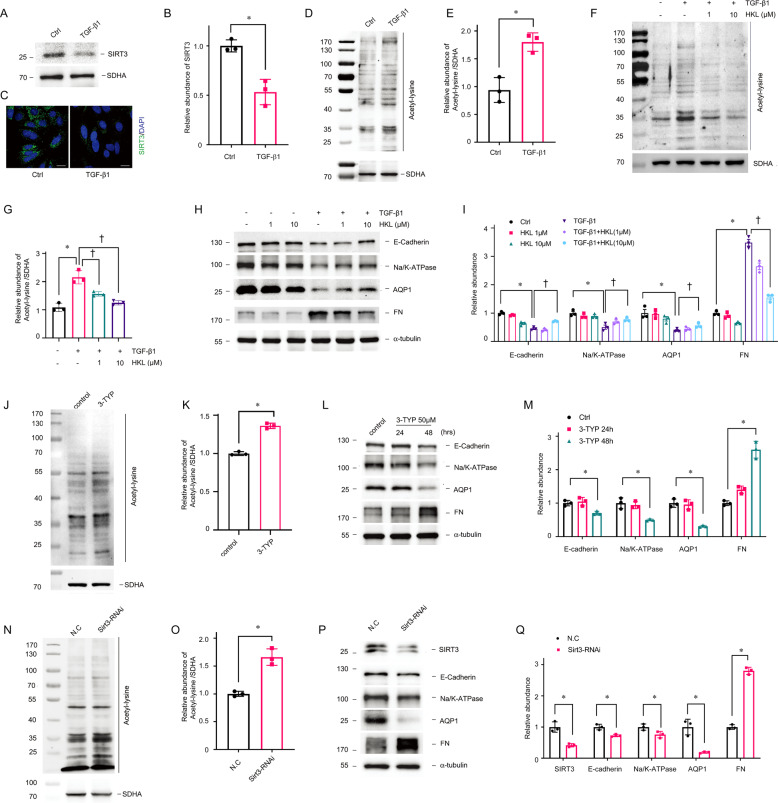
SIRT3 regulates TGF-β1 induced mitochondrial hyper-acetylation in TECs. Primary TECs were incubated with TGF-β1 (5 ng/mL). **A**, **B** Western blots of SIRT3 expression after incubation with TGF-β1 for 1 h. α-tubulin served as the standard (*P < 0.05; *n* = 3). **C** Representative findings of immunofluorescent staining for SIRT3 in TECs incubated with TGF-β1. The bar indicates 20 μm. **D**, **E** Acetylation in mitochondrial lysates from TECs incubated with TGF-β1 for 1 h. SDHA served as the standard (*P < 0.05; *n* = 3). **F**, **G** Western blots of acetylated lysine in mitochondrial lysates from TECs incubated with HKL then TGF-β1 stimulation for 1 h. SDHA served as the standard (*P < 0.05, ^†^*P* < 0.05; *n* = 3). **H**, **I** Western blots of E-cadherin, Na/K-ATPase, AQP1, and FN in TECs incubated with HKL then stimulated with TGF-β1 for 48 h. α-tubulin served as the standard (*P < 0.05, ^†^*P* < 0.05; *n* = 3). **J**, **K** Western blots of acetylated-lysine in mitochondrial lysates from TECs incubated for 15 min with SIRT3 selective inhibitor, 3-TYP (50 μM). SDHA served as the standard (*P < 0.05; *n* = 3). **L**, **M** Western blots of E-cadherin, Na/K-ATPase, AQP1, and FN in TECs incubated with 3-TYP (50 μM) for 24 and 48 h. α-tubulin served as the standard (*P < 0.05, ^†^*P* < 0.05; *n* = 3). **N**, **O** Western blots of acetylated lysine in mitochondrial lysates from TECs transfected with *Sirt3* siRNA compared with negative control (NC). SDHA served as the standard (*P < 0.05; *n* = 3). **P**, **Q** Western blots of SIRT3, E-cadherin, Na/K-ATPase, AQP1, and FN in TECs transfected with NC and *Sirt3* siRNA. α-tubulin served as the standard (*P < 0.05; *n* = 3).

Transforming growth factor-β (TGF-β) is the master regulator of renal fibrosis [29]. We next explored whether SIRT3 mediates mitochondrial acetylation in proximal TECs in response to TGF-β. We found that incubating primary TECs with TGF-β1 for 1 h resulted in decreased SIRT3 expression and increased mitochondrial acetylation (Fig. 4A–E). Consistent with the result in vivo, SIRT3 activation by HKL also reduced mitochondrial acetylation and blocked phenotypic changes in TECs induced by TGF-β1 (Fig. 4F–I). We blocked SIRT3 with 3-(1H-1,2,3-triazol-4-yl)pyridine (3-TYP) [30, 31] and assessed the effects of disrupting SIRT3-regulated mitochondrial acetylation. 3-TYP exerted the same phenotypic transformation as TGF-β1 described above (Fig. 4J–M). We investigated the effects of damping *Sirt3* expression with small interfering (si) RNA against *Sirt3* on acetylation, to determine the role of SIRT3 in acetylation. The reduced SIRT3 expression in TECs resulted in increased mitochondrial acetylation (Fig. 4N–O) and induced TEC injury (Fig. 4P–Q), which coincided with the effects of 3-TYP. These data in vitro showed that SIRT3 mediates the increased mitochondrial acetylation in TECs associated with pro-fibrotic stimulation.

### Pro-fibrotic stimulation substantially alters tubular mitochondrial protein acetylation

**Fig. 5:**
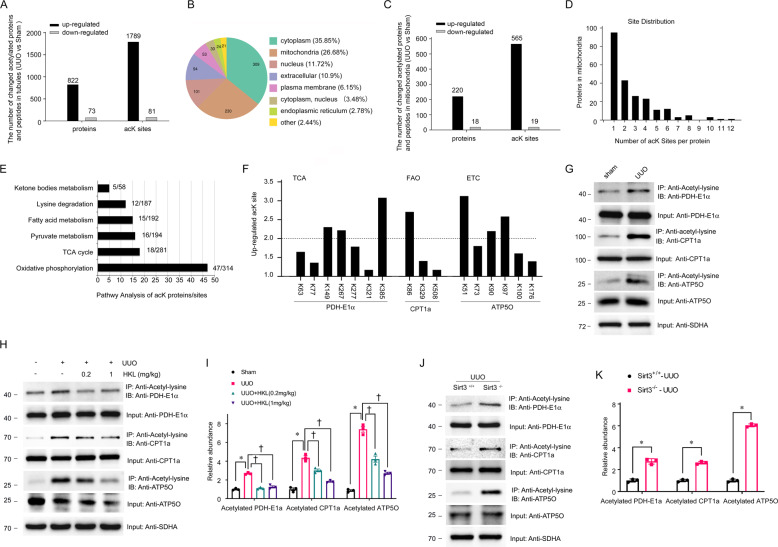
Loss of SIRT3 results in mitochondrial acetylation in renal fibrosis. **A** Numbers of upregulated and downregulated acetylated proteins and peptides (UUO vs. Sham). Sites were screened based on twofold change and *p* < 0.05 in *t* tests. **B** Subcellular classification of changed acetylated proteins. **C** Numbers of upregulated or downregulated acetylated mitochondrial proteins and peptides (UUO vs. Sham). **D** Distribution of changed AcK sites per mitochondrial protein. **E** Pathway of mitochondrial acetylome altered in UUO mice with numbers of proteins and peptides per pathway. **F** Acetylation profiles of PDHE1α from TCA cycle, CPT1a from FAO, and ATP5O from ETC. **G** Tubular mitochondria extracts from UUO and sham-operated mice on day 1 immunoprecipitated with anti-acetylated-lysine antibody and analyzed using anti-PDHE1α, anti-CPT1a, and anti-ATP5O. **H**, **I** Tubular mitochondria extracts from UUO and sham-operated mice injected with or without HKL on POD 1 were immunoprecipitated with anti-acetyl-lysine antibody and analyzed with anti-PDHE1α, anti-CPT1a, and anti-ATP5O. PDHE1α or CPT1a or ATP5O served as the standard (*P < 0.05, ^†^*P* < 0.05; *n* = 3). **J**, **K** Tubular mitochondria extracts from *Sirt3* KO and WT mice with UUO on POD 1 immunoprecipitated with anti-acetyl-lysine antibody and analyzed using anti-PDHE1α, anti-CPT1a, and anti-ATP5O. PDHE1α or CPT1a or ATP5O served as the standard (*P < 0.05; *n* = 3).

We next examined acetylome and proteome responses to fibrosis in tubules. A comparison of the tubular acetylome of mice with and without UUO (Supplementary Fig. 3A) found that 1870 of 7059 acetylation sites in 895 of 1863 proteins changed in UUO mice (Supplementary Fig. 3B). Most proteins were multi-acetylated with 58.03% having two or more AcK sites (Supplementary Fig. 3C). These findings considerably exceeded previous estimations of acetylated proteins and sites in the kidneys [32–34]. Most of the changed proteins were hyper-acetylated and in fact, acetylation at 1789 sites in 822 proteins increased more than two-fold in mice with UUO (Fig. 5A). We evaluated these proteins according to their GO cellular compartment annotations to determine the subcellular localization of the modified proteins. Most of the altered acetylated proteins resided in the cytoplasm, which contained 35.85% of all changed proteins. Mitochondria accounted for 26.68% of the changed acetylated proteins (Fig. 5B). We analyzed the altered acetylated proteins in mitochondria from fibrotic kidneys. The protein acetylation profiles in mitochondria were similar to the global changes. We found that 565 sites in 220 of 238 (92.44%) changed mitochondrial proteins were increased >2-fold and accounted for 96.91% of all acetylated sites (*P* < 0.01) (Fig. 5C). The mitochondrial proteins were multi-acetylated with 57.4% having at least two AcK sites (Fig. 5D). Pathway enrichment analysis of the hyper-acetylated mitochondrial proteins revealed that the main pathways were regulated by lysine acetylation such as oxidative phosphorylation (313 sites in 47 proteins), the TCA cycle (281 sites in 18 proteins), pyruvate metabolism (194 sites in 16 proteins), fatty acid metabolism (192 sites in 15 proteins), lysine degradation (187 sites in 12 proteins), and ketone body metabolism (58 sites in 5 proteins) (Fig. 5E, Supplementary Fig. 3D). Because metabolic activities in proximal TECs rely primarily on mitochondrial FAO to generate ATP for energy. We identified lysine acetylation sites on mitochondrial proteins involved in most of the enzymatic processes in mitochondrial metabolic pathways (Supplementary Fig. 3E). We then examined acetylation sites in proteins involved in the TCA cycle, FAO, and ETC. We found that three sites in PDHE1α in the TCA cycle, one in carnitine palmitoyltransferase 1 (CPT1) and three in ATP synthase subunit O (ATP5O) in the ETC were increased >2-fold (*P* < 0.01) (Fig. 5F). To further confirm the acetylation of mitochondrial proteins, we immunoprecipitated tubular mitochondrial lysates isolated from sham-operated and UUO mice using an anti-AcK antibody and western blotted them against PDHE1α, CPT1a, and ATP5O. The findings confirmed that mitochondrial proteins in the tubules from the fibrotic kidneys were hyper-acetylated (Fig. 5G). These data overall showed that pro-fibrotic stimulation results in the hyperacetylation of mitochondrial proteins associated with metabolic pathways.

### Mitochondrial metabolism is regulated by SIRT3 via acetylation

Previous findings have suggested that SIRT3 regulates the acetylation of mitochondrial proteins. We further investigated the acetylation of PDHE1α, CPT1a, and ATP5O in tubules from mice treated with HKL and *Sirt3* KO mice. HKL blocked the hyperacetylation of PDHE1α, CPT1a, and ATP5O in mice with UUO (Fig. 5H, I). On the contrary, *Sirt3* KO mice with or without UUO had more acetylated PDHE1α, CPT1a, and ATP5O than WT mice (Fig. 5J, K, Supplementary Fig. 2C). Taken together, these results indicate that SIRT3 can counteract the effects of acetylation on mitochondrial metabolism during the progression of renal fibrosis.

### SIRT3 regulates PDH enzymatic activity via PDHE1α deacetylation

**Fig. 6:**
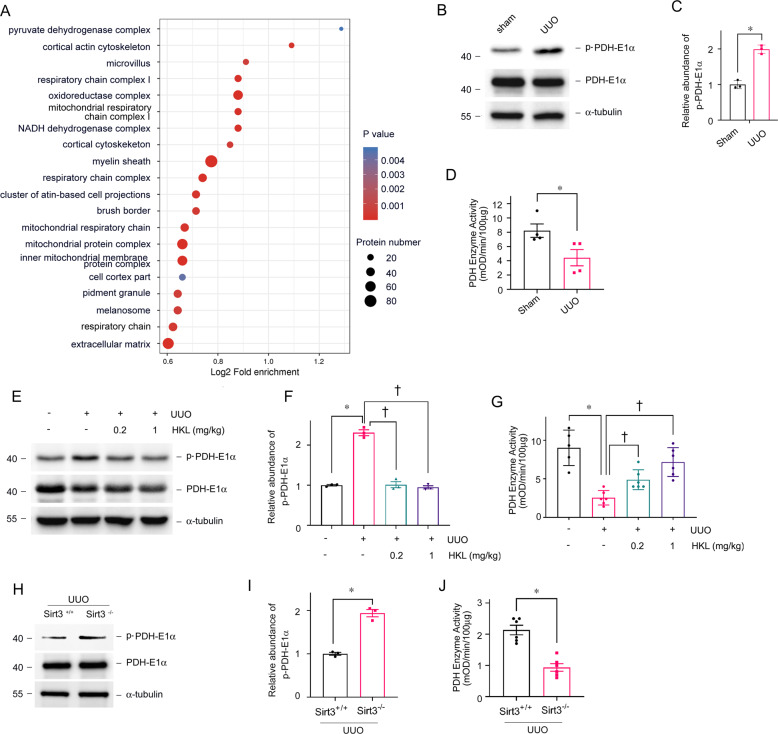
Acetylation of PDHE1α is increased in tubules from fibrotic kidneys. **A** Cellular component GO enrichment analysis of upregulated acetylated proteins in tubules separated from UUO and sham-operated mice. Bubble chart shows top 20 significantly enriched terms (vertical axis, functional category; horizontal axis, Log2 transformed ratio of upregulated acetylated proteins to identified proteins. Colored circles indicate significant *p* values; circle size indicates a number of differential proteins in terms. **B**, **C** Western blots of phosphorylated PDHE1α and total PDHE1α expression in tubules from UUO and sham-operated mice on POD 1. PDHE1α served as the standard (*P < 0.05; *n* = 3). **D** Enzyme activity of PDH in tubular mitochondria from UUO and sham-operated mice on POD 1 (*P < 0.05 vs. sham; *n* = 4); **E**, **F** Western blots of phosphorylated PDHE1α and total PDHE1α expression in tubules from UUO and sham-operated mice injected with or without HKL on POD 1. PDHE1α served as the standard (*P < 0.05, ^†^*P* < 0.05; *n* = 3). **G** Enzyme activity of PDH in tubular mitochondria from UUO and sham-operated mice injected with or without HKL on POD 1. (*P < 0.05, ^†^*P* < 0.05; *n* = 5–6). **H**, **I** Western blots of phosphorylated PDHE1α and total PDHE1α expression in tubules from *Sirt3* KO and WT mice with UUO on POD 1. PDHE1α served as the standard (*P < 0.05; *n* = 3). **J** Enzyme activity of PDH in tubular mitochondria from *Sirt3* KO and WT mice with UUO on POD 1. (*P < 0.05; *n* = 6).

Cellular component GO enrichment analysis of upregulated acetylated proteins in tubules collected from mice with UUO revealed that pyruvate dehydrogenase complex (PDHC) was the most obviously enriched GO term (Fig. 6A). We previously immunoprecipitated tubular mitochondrial lysates using anti-AcK and found hyperacetylated PDHE1α in fibrotic kidneys (Fig. 5G). The results of several studies have suggested that PDHE1α acetylation results in PDHE1α phosphorylation and the inhibition of pyruvate dehydrogenase complex (PDC) activity [35]. The enzyme activity of PDH and western blots of proteins in tubules separated from fibrotic kidneys also confirmed that increased lysine PDHE1α acetylation was accompanied by reduced PDH enzyme activity and increased PDHE1α phosphorylation (Fig. 6B–D). Activation SIRT3 by HKL could inhibit PDHE1α phosphorylation and restore PDH enzyme activity in mice with UUO (Fig. 6E–G). On the contrary, *Sirt3* KO mice with UUO had more PDHE1α phosphorylation and reduced PDH enzyme activity (Fig. 6H–J).

**Fig. 7:**
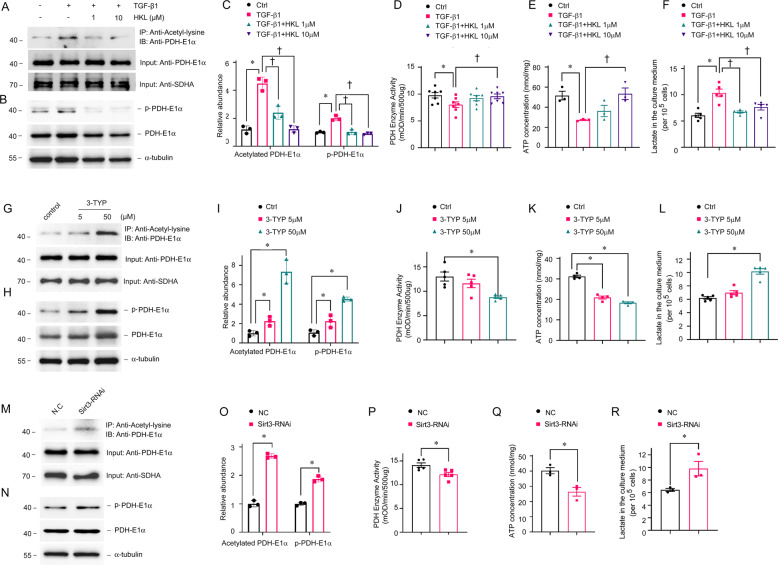
SIRT3 regulates PDHE1α acetylation and PDH enzyme activity in tubular epithelial cells. TECs incubated with or without HKL then stimulated with TGF-β1 for 1 h (**A**–**F**), TECs stimulated with 3-TYP for 15 min (**G**–**L**), and TECs transfected with *Sirt3* siRNA (**M**–**R**) for 12 h. Mitochondrial lysates immunoprecipitated with anti-acetyl-lysine antibody and analyzed with anti-PDHE1α. (**A**, **C**, **G**, **I**, **M**, **O**). PDHE1α served as the standard (*P < 0.05, ^†^*P* < 0.05; *n* = 3). Western blots of phosphorylated and total PDHE1α expression in TEC lysates (**B**, **C**, **H**, **I**, **N**, **O**). PDHE1α served as the standard (*P < 0.05, ^†^*P* < 0.05; *n* = 3). Enzyme activity of PDH in TECs incubated with or without HKL, then stimulated with TGF-β1 (*P < 0.05 vs. control, *n* = 7; ^†^*P* < 0.05 vs. TGF-β1, *n* = 7) (**D**), or 3-TYP (*P < 0.05 vs. vehicle, *n* = 5) (**J**), or transfected with *Sirt3* siRNA (*P < 0.05 vs. NC siRNA transfection, *n* = 5) (**P**); levels of ATP in TECs incubated with or without HKL, then stimulated with TGF-β1 (*P < 0.05 vs. control, *n* = 3; ^†^*P* < 0 .05 vs. TGF-β1 stimulation, *n* = 3) (**E**), or 3-TYP (*P < 0.05 vs. vehicle, *n* = 4) (**K**), or transfected with *Sirt3* siRNA (*P < 0.05 vs. NC siRNA transfection, *n* = 3) (**Q**); lactate levels in TECs incubated with or without HKL, then stimulated with TGF-β1 (*P < 0.05 compared with control, *n* = 3; ^†^*P* < 0.05 compared with TGF-β1 stimulation, *n* = 3) (**F**), or by 3-TYP stimulation (*P < 0.05 compared with vehicle treatment, *n* = 3) (**L**), or by *Sirt3* siRNA transfection (*P < 0.05 compared with NC siRNA transfection, *n* = 3) (**R**).

We investigated the role of SIRT3 on the regulation of PDHE1α acetylation in TECs incubated with TGF-β1 in vitro. We found that increased PDHE1α acetylation was accompanied by reduced PDH enzyme activity and increased PDHE1α phosphorylation (Fig. 7A–D). The activation of SIRT3 by HKL repressed PDHE1α acetylation, and HKL restored phosphorylated PDHE1α, PDH enzyme activity, ATP levels, and lactate production (Fig. 7A–F). We inhibited SIRT3 activity using 3-TYP and inhibited SIRT3 expression via *Sirt3* siRNA transfection. Each of 3-TYP or *Sirt3* siRNA transfection increased PDHE1α acetylation, increased PDHE1α phosphorylation, reduced PDH enzyme activity, decreased ATP levels, and increased lactate production (Fig. 7G–R). These data support the notion that SIRT3 functions in PDHE1α deacetylation and regulates a metabolic switch in TECs during the development of renal fibrosis.

### Sites where acetylation regulates PDHE1α activity

**Fig. 8:**
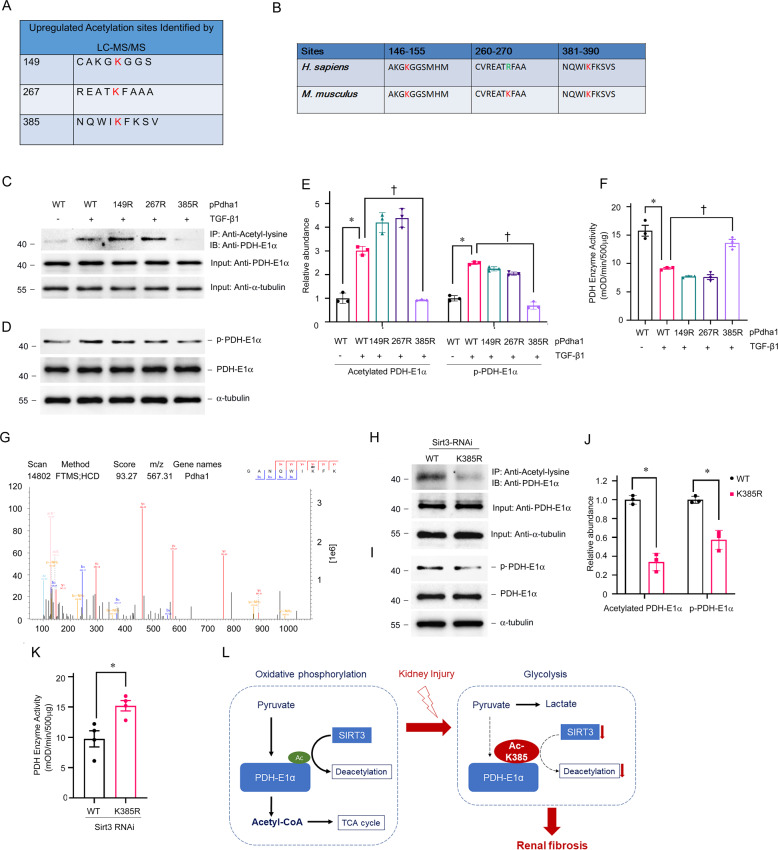
Discovery of acetylated sites of PDHE1α as a target of SIRT3 in TECs. **A** Summary of peptide fragments of upregulated acetylated lysine residues of PDHE1α; **B** Alignment of sequences around K149, K267, K385 of human and mouse PDHE1α; **C**–**E** TECs transfected with expression vectors for WT PDHE1α, PDHE1α single acetylation-point mutant K149R, K267R, or K385R, then stimulated with TGF-β1. Levels of acetylated PDHE1α (**C**, **E**), phosphorylated PDHE1α (**D**, **E**), and PDH enzyme activity (**F**) in cell lysates. PDHE1α served as the standard (*P < 0.05, ^†^*P* < 0.05; *n* = 3). **G** Representative *m*/*z* spectra (K385) determined from mass spectrometry analyses of PDHE1α. **H**–**K** TECs transfected with *Sirt3* siRNA followed by expression vectors for WT PDHE1α or PDHE1α K385R. Mitochondrial lysates immunoprecipitated with anti-acetyl-lysine antibody and analyzed with anti-PDHE1α. PDHE1α served as the standard (*P < 0.05; *n* = 3) (**H**, **J**); Western blots of phosphorylated PDHE1α and total PDHE1α in TECs in *Sirt3* knockdown TECs transfected with WT or PDHE1α K385R. PDHE1α served as the standard (*P < 0.05; *n* = 3) (**I**, **J**). **K** Enzyme activity of PDH in *Sirt3* knockdown TECs with PDHE1α K385R transfection vs. WT (*P < 0.05 vs. WT transfection, *n* = 4). **L** Diagram depicts the role of SIRT3 in the control of PDHE1α deacetylation in renal fibrosis.

Mass spectrometry-based proteomic analyses have identified three potentially acetylated sites in PDHE1α (Fig. 8A). The K149 and K385 of PDHE1α are conserved from mice to humans (Fig. 8B). We generated an acetyl-deficient K-R mutant of PDHE1α at K149 (149R), K267 (267R), and K385 (385R) to understand more about these acetylation sites. Western blotting with anti-AcK antibody showed that K385 was the major acetylation site of PDHE1α in TECs incubated with TGF-β1 (Fig. 8C, E). The activity of PDH and PDHE1α phosphorylation remained unchanged in TECs with the K385R mutation stimulated with TGF-β1 (Fig. 8D, F). Figure 8G shows the MS/MS spectrum of the acetylated K385 site. We then explored whether SIRT3 regulates acetylation at the K385 site. Wildtype (WT) and 385R mutant plasmid of PDHE1α were transfected with *Sirt3* siRNA in TECs. K385 mutation damped the acetylation of PDHE1α induced by *Sirt3* knockdown (Fig. 8H, J). The PDH activity was higher and PDHE1α phosphorylation was lower in TECs with K385 mutation (Fig. 8I–K). These results suggest that the acetylation of lysine 385 in PDHE1α regulated by SIRT3 represents a key element in the function of PDH in TECs under pro-fibrotic stimulation (Fig. 8L).

## Discussion

To our knowledge, the present study is the first to analyze the spectrum of the acetylome of TECs in fibrotic kidneys and show that most mitochondrial proteins were hyper-acetylated. Our findings support the notion that SIRT3 is a crucial regulator of mitochondrial protein acetylation and metabolic homeostasis. The deacetylation of PDHE1α by SIRT3 provides the primary link between glycolysis and the TCA cycle in TECs during the development of renal fibrosis.

Renal TECs are rich in mitochondria, which rely on oxidative phosphorylation (OXPHOS) to generate ATP. Reduced OXPHOS in proximal TECs contributes to AKI, DN and interstitial fibrosis [10]. Lysine acetylation is a novel post-translational modification of energy metabolism. Numerous enzymes involved in energy metabolism are acetylated in many metabolic diseases [36]. The discovery of hyper-acetylated renal mitochondrial protein provides a new avenue for understanding mitochondrial dysfunction in the development of renal fibrosis. We found that 1789 unique AcK sites in 822 proteins were hyper-acetylated in TECs from UUO, compared with sham-operated mice. Mitochondrial proteins are highlighted as prominent targets of tubular hyper-acetylation (~26%) in fibrotic kidneys. Almost all mitochondrial proteins involved in FAO, TCA cycle, and the ETC were hyper-acetylated. Similar acetylation profiles have been identified in diabetic kidneys and ethanol-induced nephropathy [33, 34]. Acetylation neutralizes the positive charge of lysine and impairs the catalytic activity of most mitochondrial enzymes. Acetylation reduces the activity of long-chain acyl-CoA dehydrogenase that catalyzes the first FAO in mitochondria and results in the excessive accumulation of acylcarnitines and triglycerides [37, 38]. The acetylation of proteins such as PDHE1α, ATP5O, and CPT1a in the TCA cycle and the ETC results in reduced activity and disrupted oxidative phosphorylation, decreased ATP production, aberrant lipid deposition, and increased glycolysis indicated dysfunctional mitochondrial metabolism in tubules from fibrotic kidneys.

The major mitochondrial protein deacetylase is SIRT3. Mitochondrial proteins were remarkably hyper-acetylated in mice lacking SIRT3 [39–41]. The expression of SIRT3 was decreased in a time-dependent manner after UUO. However, mitochondrial acetylation levels were increased sharply after UUO, peaked at day 1, and then gradually decreased. TECs are rich in mitochondria, which is the main source of acetyl-CoA [7]. The decrease of acetylation level at day 7 after UUO may be due to tubular atrophy and mitochondrial dysfunction [42, 43].

Acetylome and target protein analyses have shown that SIRT3 functions in the regulation of OXPHOS, FAO, and the TCA cycle. Many studies have shown that SIRT3 is expressed predominantly in mitochondria-rich tissues such as the liver, muscle, heart, brain, and kidney [44]. Furthermore, SIRT3 is prominently expressed in renal cortical tubular cells from younger mice and gradually decreases over time [45], as well as with AKI [18, 19, 46], DN [14, 23, 47], and hypertension nephropathy [48]. Targeting SIRT3 can effectively reduce mitochondrial fragmentation, reduce the degree of renal damage, and accelerate the recovery of renal function, suggesting that SIRT3 could promote recovery from kidney injury by maintaining the stability of mitochondrial structure and function [18, 19, 46]. The consequences of *Sirt3* KO in mice are that they develop fibrosis in the kidneys, liver, and lungs at the age of 15 months [25]. Pro-fibrotic stimulation reduces renal SIRT3 expression, increases mitochondrial protein acetylation, and disrupts mitochondrial metabolism. Because SIRT3 overexpression could rescue tubule damage and ameliorate renal fibrosis, we believe that reducing SIRT3 and increasing mitochondrial protein acetylation is a novel mechanism of tubular disturbance in the development of renal fibrosis.

Cellular component GO enrichment findings revealed that the PDC was the most obviously acetylated target in fibrotic kidneys. The PDC functions as a metabolic center that catalyzes pyruvate into acetyl-CoA, thus linking glycolysis to the TCA cycle. The metabolic profile of renal TECs during the development of renal fibrosis switches from OXPHOS to glycolysis, which is relevant to SIRT3 expression and activity. Lactic acid has been shown to activate myofibroblasts [49] and promote the transformation of M2 macrophages [50]. Inhibition of PDH activity leads to metabolic transformation to glycolysis, resulting in increased lactic acid production, which may promote the progressing of renal fibrosis. Thus, we postulate that reduced SIRT3 expression and hyper-acetylated PDC could be the mechanism of metabolic reprogramming in fibrotic TECs.

The PDC comprises the catalytic enzymes, PDHE1α, dihydrolipoamide transacetylase (DLAT)/E2, and dihydrolipoamide dehydrogenase (DLD)/E3. The activity of the PDC is regulated by the phosphorylation and dephosphorylation of PDHE1α catalyzed by pyruvate dehydrogenase kinases (PDK) and phosphatases (PDP). Emerging evidence suggests that lysine acetylation of PDHE1α contributes to the inhibitory regulation of PDC. The K63, K244, K267, K267, K313, K321, and K336 sites in PDHE1α have been posted on UniProt (https://www.uniprot.org). Lysine 83 [51], 321 [52, 53], and 336 [35] play functional roles in PDHE1α acetylation. Mass spectrometry-based-proteomic analyses have shown that multiple lysine residues in PDHE1α are acetylated in the kidneys. We found that K149, K267, and K385 in PDH were significantly hyper-acetylated in fibrotic kidneys. We used K149R, K267R, and K385R mutations to determine whether acetylation at K149, K267, or K385 could alter the catalytic function of PDH. We found that K385R was resistant to TGF-β1 and that *Sirt3* knockdown resulted in the inhibition of PDH enzyme activity. These results suggested that SIRT3 regulates PDHE1α activity via lysine 385 deacetylation in TECs.

In summary, our data showed that mitochondrial proteins involved in regulating energy metabolism were acetylated and targeted by SIRT3 in TECs. The deacetylation of PDHE1α at lysine 385 by SIRT3 plays a key role in metabolic reprogramming in renal fibrosis.

## Materials and methods

### Human kidney tissue handling

We collected biopsy specimens from patients at the kidney disease center of the Second Affiliated Hospital of Nanjing Medical University. All patients provided written informed consent to participate in the study, the protocol of which was implemented in accordance with the 1975 Declaration of Helsinki (2013 amendment), and approved by the Medical Experiment Ethics Committee at the Second Affiliated Hospital of Nanjing Medical University (2015KY018). The patients were assigned to groups based on whether they had <20% or ≥20% fibrotic areas determined by Masson staining. The kidney biopsy samples were further analyzed by qRT-PCR and immunohistochemical staining for SIRT3 (cat: 2627, Cell Signaling Technology [CST] Inc., Danvers, MA, USA) and acetyl-lysine (cat: 9441, CST). Total RNA of human kidney biopsy sample was prepared using RNeasy FFPE Kits (cat: 73504, Qiagen GmbH, Hilden, Germany).

### Mice and animal models

Male C57BL/6J mice purchased from Nanjing Medical University Experimental Animal Center were housed according to the guidelines of the Institutional Animal Care and Use Committee at Nanjing Medical University. Renal fibrosis was induced in mice in vivo by UUO. HKL was administered intraperitoneally (i.p.) to UUO mice at doses of 0.2 and 1 mg/kg body weight (bw) for 1 or 7 consecutive days.

The *Sirt3* KO mice were provided by Professor Jiahe Xie (Gannan Medical University, Jiangxi, China). Male *Sirt3* KO mice and littermate WT mice were used in experiments at the age of 6 weeks. Renal injury was generated by UUO in *Sirt3* KO and WT mice. All animals were randomly assigned into different groups (n: 5–8). No blinding was done.

### Cell culture and treatment

Primary TECs were separated and cultured as described [54, 55]. Recombinant human TGF-β1 (cat: 100-B-010-CF, R&D Systems, USA), HKL (cat: S2310, Selleck Chemicals, USA), and 3-TYP (cat: HY-109331, MCE, China) were added to serum-free medium for various periods. TECs were transfected with *Sirt3* siRNA (Ibsbio, Shanghai, China) using Transfectamine RNAiMAX (Invitrogen, Carlsbad, CA, USA), and Pdha1 WT, K149R mutant, K267R mutant, K385R mutant plasmids were transfected into TECs using Lipofectamine 2000 reagent (Invitrogen, USA) as described by the manufacturer.

### Mitochondria isolation

Mitochondria isolated from mouse renal tubular using the Tissue Mitochondria Isolation Kit (cat: C3606, Beyotime, China), and mitochondria of cultured PTCs were isolated by using the Cell Mitochondria Isolation Kit (cat: C3601, Beyotime) according to the manufacturer’s protocol.

### Western blot analysis

Proteins from cultured PTCs or kidneys or mitochondria were separated by sodium dodecyl sulfate-polyacrylamide gel electrophoresis (SDS-PAGE) and transferred onto polyvinylidene difluoride membranes. The blots were incubated with anti-SIRT3 (cat: 5490, CST), anti-Acetyl-lysine (cat: ab22550, Abcam, Cambridge, UK), anti-E-Cadherin (cat: 610181, BD Biosciences, San Jose, CA, USA), anti-Na/K-ATPase (cat: 3010, CST; ab76020, Abcam), anti-AQP1 (cat: AB2219, Millipore, Billerica, MA, USA), anti-FN (cat: F3648, Sigma Aldrich, St. Louis, MO, USA), anti-Collagen I (cat: 1310-01, Southern Biotech, Birmingham, AL, USA), anti-PDH-E1α (cat: ab110334, Abcam), anti-p-PDH (Ser293)(cat: ab92696, Abcam), anti-CPT1a (cat: ab128568, Abcam), anti-ATP5O (cat: ab110276, Abcam), anti-α-tubulin (cat: T9026, Sigma Aldrich), and anti-SDHA (cat: 11998, CST). Quantification was performed by measuring the intensity of the signals with the aid of the National Institutes of Health Image J software package.

### Immunoprecipitation and immunoblotting

The isolated mitochondria were resuspended in RIPA buffer in the presence of protease inhibitors. For immunoprecipitation, preclear lysate by adding appropriate control IgG (normal rabbit IgG, cat: 12–370, Millipore), together with Protein A/G PLUS-Agarose (cat: sc-2003, Santa Cruz, Biotech, CA, USA), incubate at 4 °C for 1 h. After centrifuging, transfer supernatant to a fresh microcentrifuge tube. Add PDH-E1α antibody (cat:ab168379, Abcam) or acetyl-lysine antibody (cat: 9814, CST), incubate at 4 °C overnights. Add Protein A/G PLUS-Agarose and incubate at 4 °C for 4 h, collect immunoprecipitants by centrifugation and discard the supernatant. Wash pellet 4 times with PBS buffer, bound proteins were eluted by boiling in SDS sample buffer, resolved by SDS-PAGE, and then subjected to Western analysis.

### PDH activity assay

PDH activity was measured using the PDH Enzyme Activity Assay Kit (cat: ab109902, Abcam) according to the manufacturer’s protocol.

### Lactate, pyruvate, ATP assay

Lactate concentration was measured with the Lactate Assay Kit (cat: K607, BioVision, Mountain View, CA, USA), and Pyruvate concentration was measured with the Pyruvate Assay Kit (cat: K609, BioVision). ATP concentration was measured by using the ATP Assay Kit (cat: S0026, Beyotime).

### Histology, immunohistochemistry, and Immunofluorescent staining

H&E and Masson staining, immunohistochemical and immunofluorescence staining were performed according to an established procedure. The following primary antibodies for immunohistochemical and immunofluorescence staining were used: anti-Acetyl-lysine (cat: 9441, CST), anti-SIRT3 (cat: 2627, CST; cat: 365175, Santa Cruz), anti-Na/K-ATPase (cat: sc-28800, Santa Cruz), anti-FN (cat: F3648, Sigma Aldrich), and anti-Collagen I (cat: 1310-01, Southern Biotech).

### LC–MS/MS analysis

Tubules separated from mice under sham- or UUO-operation on POD 1 were ground by liquid nitrogen for acetylome analysis perform by PTM-Biolabs (HangZhou, China). The Acetylation quantitative study was carried out Acetylation enrichment method and high-resolution liquid chromatography–mass spectrometry (LC–MS)/MS. Normalization with protein quantification to remove the effect of protein expression on modification abundance was used for subsequent bioinformatics analysis. Screening of the differentially modified sites followed the following criteria: twofold change threshold, *t* test *p* value < 0.05.

The mass spectrometry proteomics data have been deposited to the ProteomeXchange Consortium via the PRIDE partner repository with the dataset identifier PXD021357.

Website: http://www.ebi.ac.uk/pride

Username: reviewer_pxd021357@ebi.ac.uk

Password: f6UpWA6l

### Statistical analysis

Data are presented as means ± standard error of the mean. Data were statistically analyzed using SigmaStat software (Jandel Scientific, San Rafael, CA, USA). Between-group comparisons were assessed using one-way analyses of variance, followed by Student–Newman–Keuls tests. Values with *P* < 0.05 were considered statistically significant.

## Supplementary information

supplemental information

## Data Availability

All data generated or analyzed during this study are included in this published article and its supplementary information files. The datasets used and analyzed during the current study are available from the corresponding author on reasonable request.
