# Supplementary material for: Sirtuin 3 regulates mitochondrial protein acetylation and metabolism in tubular epithelial cells during renal fibrosis
Source: Cell Death Dis. 2021 Sep 13;12(9):847. doi: 10.1038/s41419-021-04134-4 (PMC8437958; doi:10.1038/s41419-021-04134-4)
Supplement: Supplementary file 1 — supplemental information [file 41419_2021_4134_MOESM1_ESM.docx]

**Supplemental Information**


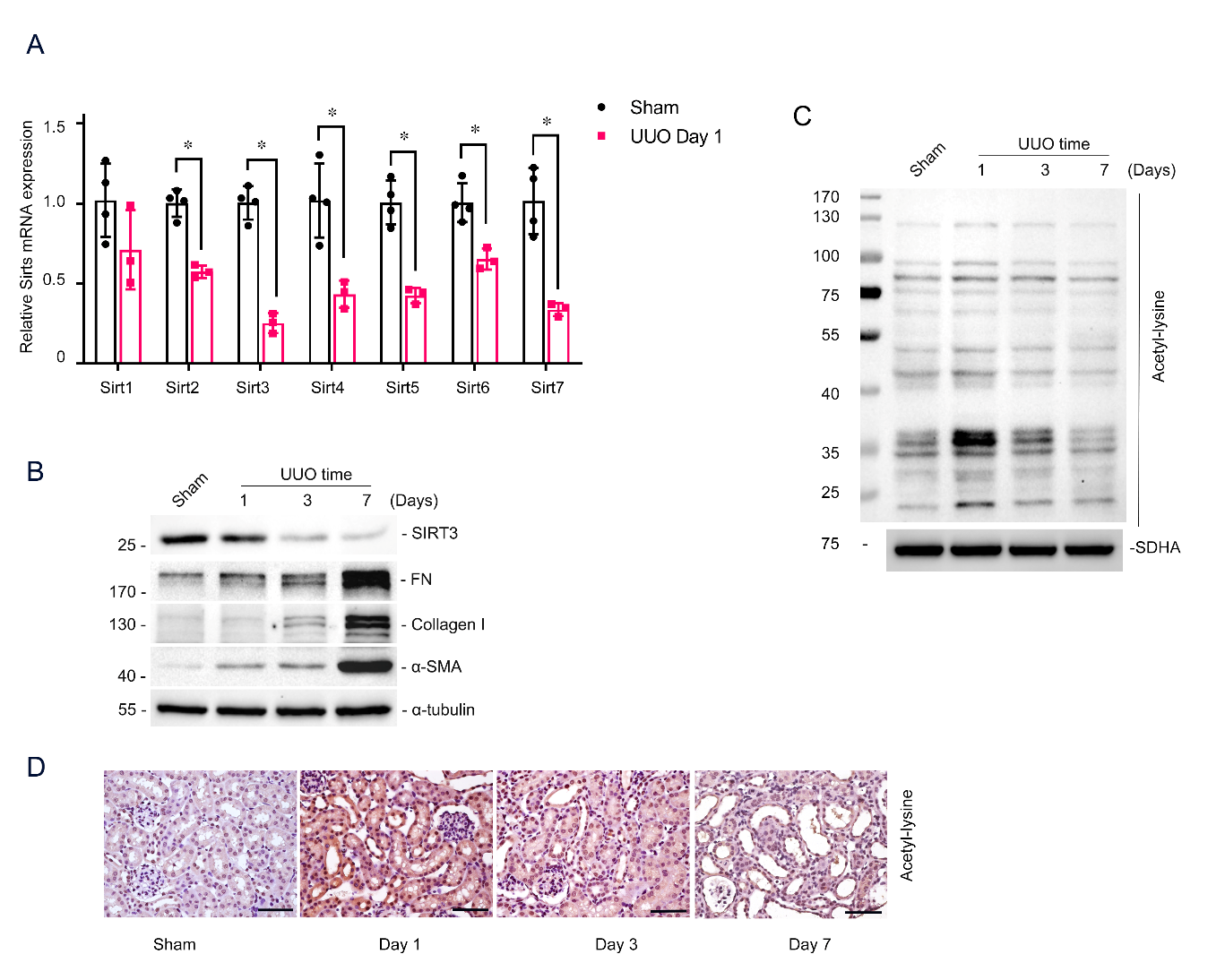


**Supplementary Figure 1: SIRT3 expression and acetyl-lysine level in tubules during fibrosis**

A: Relative *Sirt1*, *Sirt2*, *Sirt3*, *Sirt4*, *Sirt5*, *Sirt6* and *Sirt7* mRNA expression in tubules from UUO mice at day 1(**P* < 0.05; n = 3~4); B: Western blots of SIRT3, FN, collagen I and α-SMA expression in UUO mice at day 1, day 3 and day 7; C: Western blots of acetyl-lysine in tubular mitochondrial lysates from UUO mice at day 1, day 3 and day 7. D: Representative immunostaining of acetyl-lysine expression in kidneys. Bar indicates 50 μm.


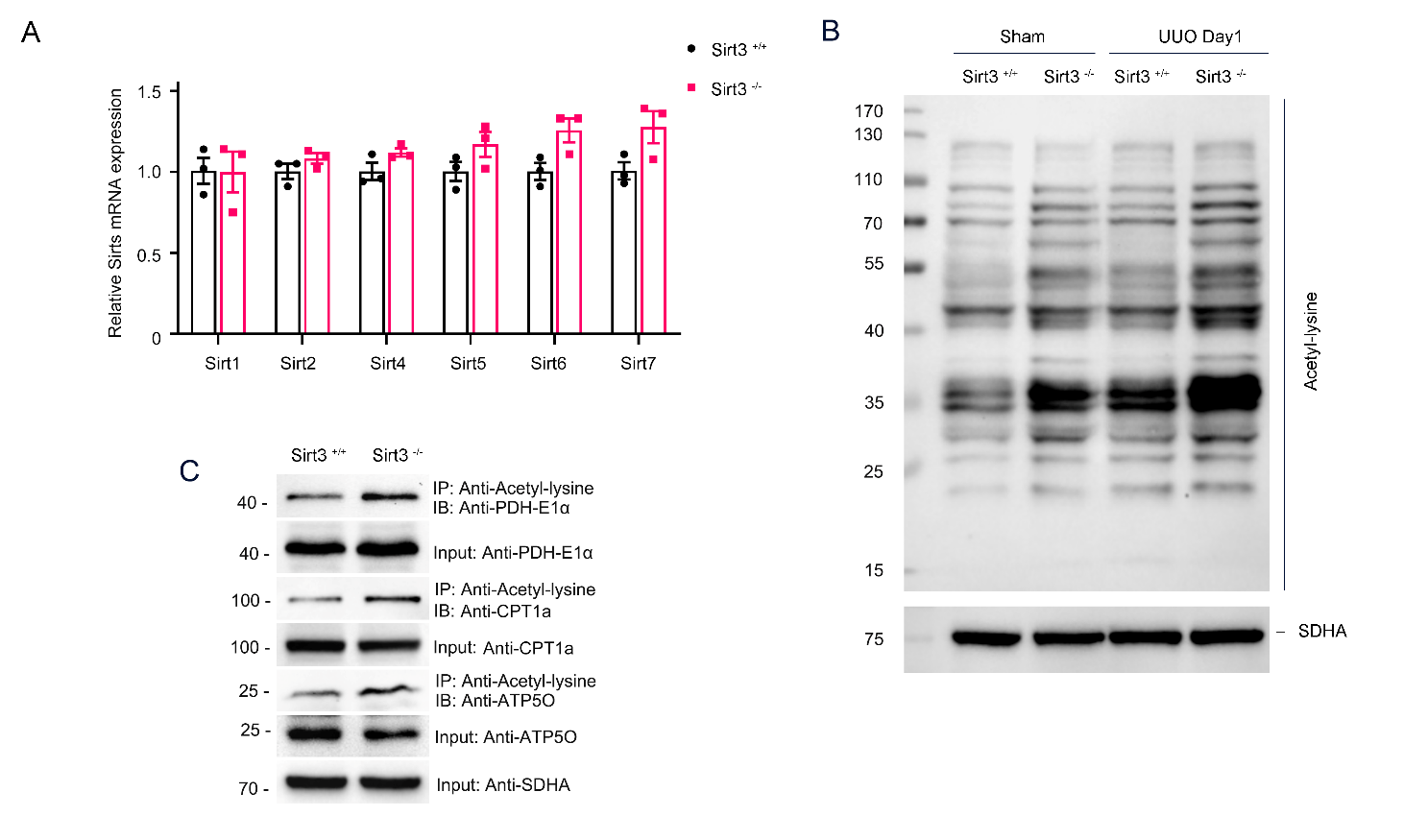


**Supplementary Figure 2: Increased acetylation of mitochondrial proteins in tubules from *Sirt3* KO mice**

A: Relative *Sirt1*, *Sirt2*, *Sirt4*, *Sirt5*, *Sirt6* and *Sirt7* mRNA expression in tubules from *Sirt3* KO mice and WT mice (n =3). B: Western blots of proteins from tubules isolated from kidneys from *Sirt3* KO mice and WT mice with or without UUO using anti-acetylated-lysine antibody. C: Tubular mitochondria extracts from *Sirt3* KO and WT mice at 6 weeks immunoprecipitated with anti-acetyl-lysine antibody and analyzed using anti-PDHE1α, anti-CPT1a and anti-ATP5O. PDHE1α or CPT1a or ATP5O served as the standard.


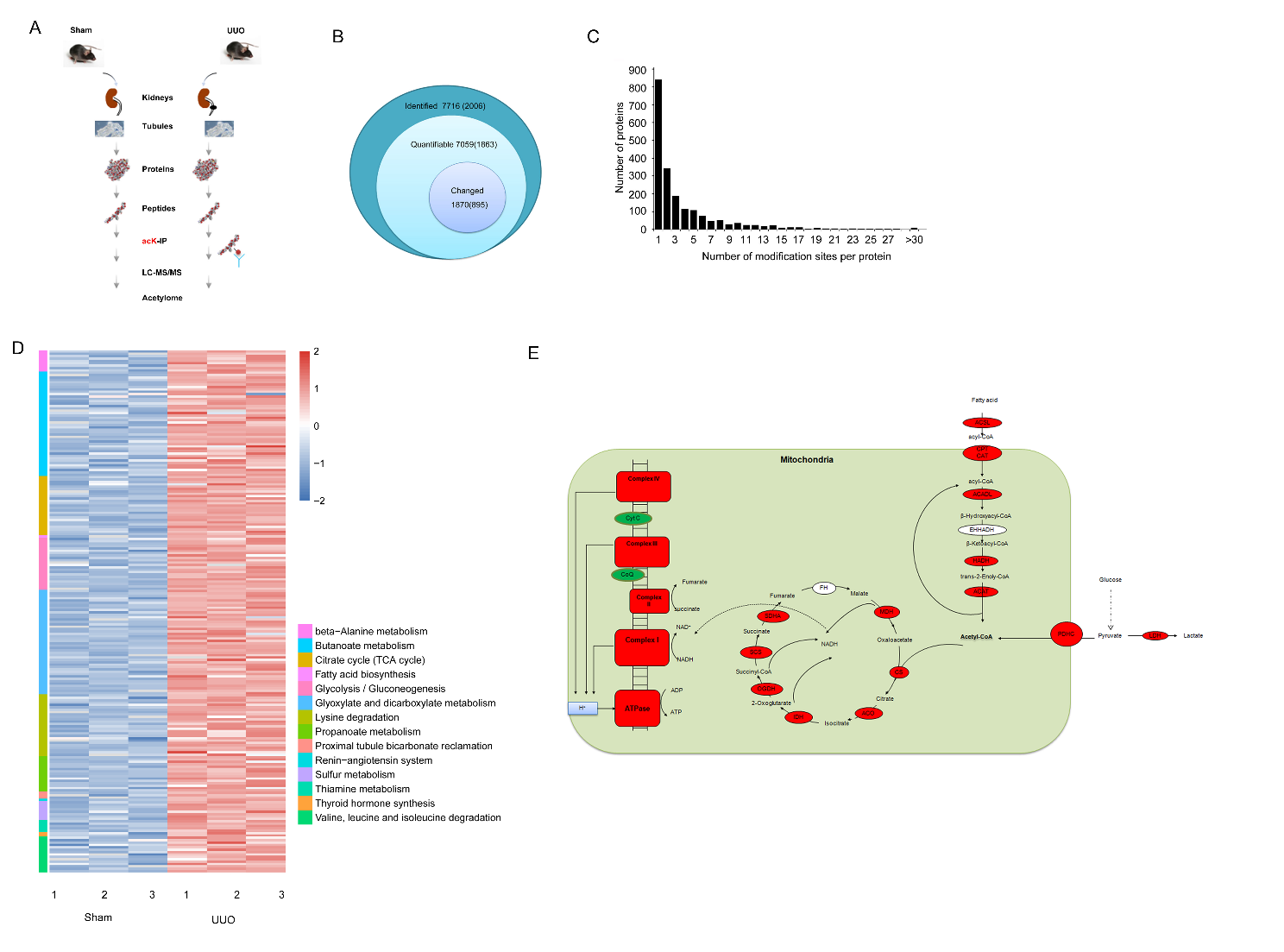


**Supplementary Figure 3. Properties of altered acetylated proteins and peptides in tubules isolated from UUO and sham -operated mice.**

A: Acetylated peptides and proteins identified by LC-MS/MS in tubules isolated from kidneys of UUO and sham-operated mice on postoperative day (POD) 1; B: Acetylated sites and proteins quantified in kidneys; C: Distribution of AcK sites per protein; D: Comprehensive heatmap of cluster analysis based on KEGG pathway in mitochondria. Horizontal axis, comparison group; vertical axis, enriched functional terms. Red to blue gradient represents *p* values of specified functional terms in comparison group. Red and blue, represent significantly and not-significantly enriched; E: Organization of acetylated proteins in mitochondrial metabolism including FAO, TCA, ETC (red, hyperacetylation).

**Supplementary Table 1: Baseline clinical characteristics of renal biopsy samples**

| **Characteristic** | **Fibrosis area (＜20%)** | **Fibrosis area (≥20%)** | **P value** |
| --- | --- | --- | --- |
| Patients (n) | 6 | 8 |  |
| Men (n) | 3 | 4 | >0.9999 |
| Age (yr) | 42.50±19.82 | 51.13±13.53 | 0.3506 |
| Systolic BP (mmHg) | 121.83±16.44 | 133.13±13.73 | 0.1863 |
| Diastolic BP (mmHg) | 77.00±12.63 | 82.00±12.64 | 0.4778 |
| Hemoglobin (g/L) | 130.00±14.63 | 129.63±13.69 | 0.9615 |
| Blood glucose (mmol/L) | 5.14±0.73 | 5.66±1.75 | 0.5484 |
| Serum creatinine (mg/dL) | 0.73±0.10 | 1.44±1.15 | 0.1654 |
| eGFR (ml/min/1.73m^2^) | 112.30±26.41 | 64.40±21.21 | 0.0027** |
| Urine albumin (g/24h) | 0.58±0.96 | 3.25±3.52 | 0.1305 |

Data are represented as mean ± SEM

BP: Blood Pressure

eGFR: [Estimated Glomerular Filtration Rate](https://www.webmd.com/a-to-z-guides/glomerular-filtration-rate)

***P*<0.01
